# Pneumatosis intestinalis with a benign clinical course: a report of two cases

**DOI:** 10.1186/s13104-017-2647-0

**Published:** 2017-07-25

**Authors:** Aya Takase, Nobuhiro Akuzawa, Hiroshi Naitoh, Jun Aoki

**Affiliations:** 1 Department of Radiology, Gunma Chuo Hospital, 1-7-13 Koun-cho, Maebashi, Gunma 371-0025 Japan; 2 Department of General Medicine, National Hospital Organization Shibukawa Medical Center, 383 Shiroi, Shibukawa, Gunma 377-0280 Japan; 3 Department of Surgery, Gunma Chuo Hospital, 1-7-13 Koun-cho, Maebashi, Gunma 371-0025 Japan

**Keywords:** *Clostridium* species, Immunotolerance, Pneumatosis intestinalis, Voglibose

## Abstract

**Background:**

Pneumatosis intestinalis (PI) is a rare condition characterized by the presence of gas within the gastrointestinal tract wall. Most cases of PI have a benign clinical course, although some have serious outcomes. Mechanical stress on or bacterial infection of the gastrointestinal tract wall may be responsible for the onset of PI, but the detailed mechanism of PI pathogenesis is still unclear. Here, we describe two Japanese patients presenting with benign PI.

**Case presentation:**

Case 1, a 37-year-old previously healthy male patient, had a 1-week history of abdominal pain, and case 2, a 78-year-old female diabetic patient, had a 2-week history of voglibose treatment and abdominal pain. Intramural gas was mainly distributed in the colon in case 1 and in the small intestine in case 2. Interestingly, neither patient showed obvious inflammatory signs upon admission and recovered spontaneously with conservative treatment, including fasting and fluid infusion without antibiotics. Voglibose treatment was terminated in case 2. Recent studies have shown the presence of nonpathogenic bacteria, such as *Clostridium* spp., in PI lesions, which usually play an important role in modulating the tolerance of the gastrointestinal immune responses. The benign clinical course and spontaneous resolution of PI in these patients, without specific treatment, suggests that nonpathogenic indigenous bacteria in the gastrointestinal tract participate in the pathogenesis of PI.

**Conclusion:**

In patients with benign PI, the absence of an inflammatory response and the spontaneous resolution of the disease without specific treatment suggest the participation of nonpathogenic indigenous bacteria of the gastrointestinal tract.

## Background

Pneumatosis intestinalis (PI) is a rare condition characterized by the presence of gas within the wall of the gastrointestinal tract, detected on imaging studies [1]. PI tends to occur most frequently in the colon in both Japanese and Chinese patients, and in patients in the United States [2–4], and may occur at any age. It displays no sex-based predominance and is most often found incidentally in asymptomatic patients during imaging studies [1]. Although most cases of PI follow a benign clinical course, other cases display serious symptoms related to intestinal ischemia or perforation, and may result in death if appropriate surgical treatment is not given [1]. The pathogenic mechanism of PI is still unclear, but various coexisting diseases, including respiratory diseases, inflammatory bowel diseases, collagen diseases, malignancies, and infectious diseases, may be associated with the onset of PI [1].

Here, we present two cases of PI. One patient showed no underlying disease, whereas the other had a history of diabetes mellitus, and developed PI 2 weeks after she was administered an α-glucosidase inhibitor (α-GI). Both cases followed a benign clinical course and were conservatively treated with only fasting and intravenous fluids. Both showed rapid improvement within 2 weeks.

## Case presentation

### Case 1

**Fig. 1:**
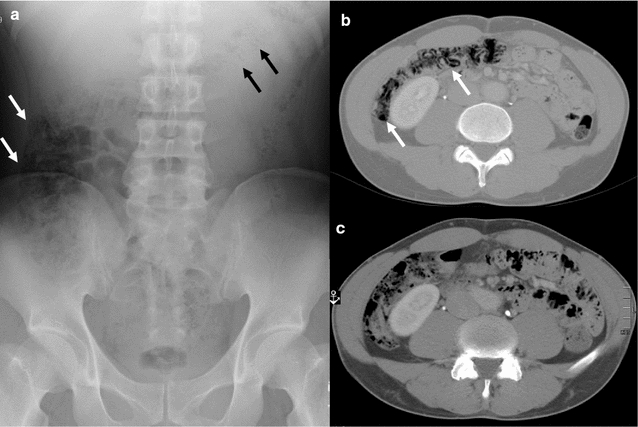
Plain abdominal radiographic and abdominal computed tomography (CT) images of case 1. **a** Plain abdominal radiograph. Small radiolucent clusters suggesting intramural air particles were observed in the ascending colon (*white arrows*), and radiolucent streaks were observed along the wall of the transverse colon (*black arrows*). **b** Abdominal CT under a wide window setting showed intramural gas in the ascending colon (*white arrow*) and transverse colon (*black arrow*). **c** Intramural colonic gases were undetectable on CT after conservative therapy for 7 days

A 37-year-old Japanese man presented with a 1-week history of abdominal pain, distension, and watery diarrhea, and was admitted to our hospital. He had no remarkable personal or family medical history, was taking no medication, and was a nonsmoker and nondrinker. The patient was 180 cm in height and weighed 75 kg, and on examination, he was afebrile (36.8 °C) with normal blood pressure (110/68 mmHg) and pulse rate (84 bpm). A physical examination revealed mild tenderness and distension of the abdomen with no signs of peritoneal irritation. Both his arterial blood O_2_ saturation (99%) and partial O_2_ pressure (99.8 Torr) were normal, and the lactic acid level in his blood was also normal (0.99 mmol/L; normal: 0.44–1.78 mmol/L). His laboratory findings, including hematology and biochemistry, were normal and showed no signs of inflammation, with a white blood cell (WBC) count of 8100/mm^3^ (normal 3500–9000/mm^3^) and a serum C-reactive protein (CRP) level of 0.08 mg/dL (normal: <0.03 mg/dL). Plain abdominal radiography showed small radiolucent clusters, suggesting intramural air particles, within the ascending colon and radiolucent streaks in the transverse colon (Fig. 1a). Computed tomography (CT) also showed intramural gas in the ascending and transverse colons (Fig. 1b). The patient was treated with fasting and intravenous fluids; no antibiotics were administered because he showed no obvious inflammatory signs upon admission. The patient’s symptoms disappeared 2 days after admission and he could take meals thereafter, with no deterioration of his physical, imaging, or laboratory findings. A stool culture upon admission produced no remarkable growth of pathogenic bacteria. CT showed that the intramural colonic gases had disappeared by day 7 (Fig. 1c) and the patient was discharged on the same day. He was followed-up for 6 months after discharge and has shown no recurrence of PI for 2 years.

### Case 2

**Fig. 2:**
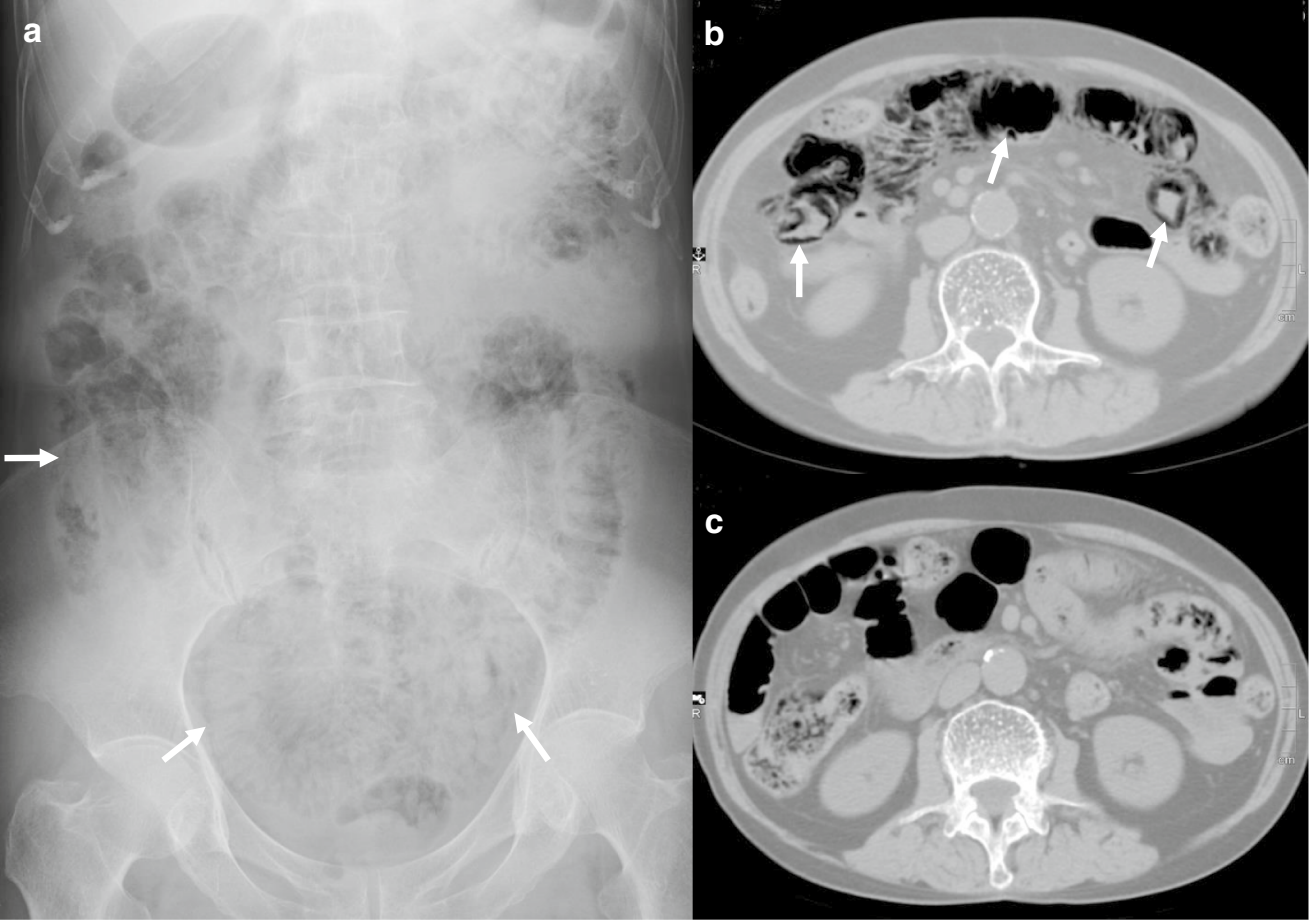
Plain abdominal radiographic and abdominal computed tomography (CT) images of case 2. **a** Plain abdominal radiograph showed several radiolucent tracks along the wall of the small intestine (*white arrows*). **b** CT of the abdomen under a wide window setting showed intramural gas widely distributed in the small intestine (*white arrows*). **c** Intramural gas in the small intestine almost disappeared after conservative therapy for 11 days

A 78-year-old Japanese woman was admitted to our hospital having suffered persistent abdominal pain for a week. She was a nonsmoker and did not consume alcohol. She had no remarkable family or personal medical history, except a diverticulectomy of the duodenum adjacent to Vater’s papilla at the age of 38 years for recurrent diverticulitis. She had been diagnosed with diabetes mellitus 3 years earlier, and had been treated with sitagliptin (50 mg/day) for 2 years. However, her blood sugar control had deteriorated and her family doctor had commenced treatment with an α-GI, voglibose (0.6 mg/day) 2 weeks before her admission. In the first several days after starting voglibose, she displayed abdominal distension and frequent flatulence, and these symptoms were gradually superseded by persistent abdominal pain and watery diarrhea without melena in the second week. Although the extent of her abdominal pain did not increase, her defecation frequency reached about 10 times a day just before admission. The patient was 158 cm in height and weighed 45 kg. At admission, her body temperature was 36.5 °C, her blood pressure was 119/81 mmHg, and her heart rate was 91 bpm and regular. A physical examination revealed tenderness of the upper abdomen on palpation, but no obvious signs of peritoneal irritation. Her arterial blood O_2_ saturation (98%) and blood gas analysis were normal. The lactic acid level in her blood was also normal (0.79 mmol/L; normal: 0.44–1.78 mmol/L). Plain abdominal radiography showed several radiolucent tracks along the wall of the small intestine (Fig. 2a). Abdominal CT also showed intramural gas widely distributed in the small intestine (Fig. 2b). Despite these imaging results, her WBC count was 4900/mm^3^ (normal: 3500–9000/mm^3^), her serum CRP level was 0.01 mg/dL (normal: <0.3 mg/dL), and her other laboratory findings showed no remarkable abnormalities, except an elevated blood glucose level of 232 mg/dL (normal: <140 mg/dL) and a hemoglobin A1c level of 6.9% (normal: <6.3%). After admission, the oral administration of both voglibose and sitagliptin was discontinued and the patient was treated with fasting and intravenous fluids, without antibiotics. The patient’s symptoms gradually decreased and had resolved completely on day 4. She remained asymptomatic and showed no deterioration after consuming meals on day 5, and she resumed treatment with sitagliptin on day 6. A stool culture on admission showed no remarkable growth of pathogenic bacteria. Abdominal CT on day 11 showed that the intramural gas in the small intestine had almost disappeared (Fig. 2c), and the patient was discharged on day 12. She has been followed-up for 12 months since discharge. The patient’s diabetes is treated with sitagliptin (50 mg/day) and ipragliflozin (50 mg/day), with good glycemic control. The discontinuation of voglibose has been maintained and the patient has shown no recurrence of PI for 1 year.

As shown in these two cases, patients with PI can follow a benign clinical course in which PI resolves without specific treatment, although there can be clinical complications in some cases, especially when underlying bowel disease is present, such as intestinal infarction or obstruction [5–7]. Abdominal CT is the most sensitive imaging technique for the diagnosis of PI [1]. A study that compared the CT findings in patients with benign or life-threatening PI showed that certain CT features, including mesenteric stranding, bowel wall thickening, and ascites, were present in more than 90% of patients with severe PI and correlated significantly with life-threatening disease [7]. Similarly, the presence of hepatoportal or portomesenteric venous gas and a linear pattern of intramural gas may predict a deleterious outcome, associated with intestinal infarction [6–8], but portal venous gas or pneumoperitoneum has been seen in a patient with benign PI [9]. In our patients, both case 1 and case 2, no obvious findings suggesting portal venous gas or pneumoperitoneum were observed. The lesion area in PI may also be important. In about half of all patients with life-threatening PI, the main PI lesion area is confined to the small intestine, whereas about 70% of patients with benign PI show colonic involvement [7]. However, it must be noted that these findings are not specific or definitive characteristics of life-threatening PI because these CT findings, including mesenteric stranding, bowel wall thickening, and ascites, are also observed in about 50% of patients with benign PI [7]. Laboratory abnormalities, including low pH (<7.3) and bicarbonate levels (<20 mEq/L), high lactate (>2 mmol/L) and amylase levels (>200 U/L), and test results consistent with disseminated intravascular coagulation, have been reported to be clinical predictors of bowel necrosis and/or mortality in patients with PI [1]. Although neither of our patients showed high-risk signs upon admission on either CT or blood tests, it is imperative that cases of life-threatening PI associated with bowel infarction are not overlooked during admission [8]. It should also be noted that nearly all the cases of PI associated with α-GI have been reported in Japan, which may reflect a higher frequency of α-GI prescription in Japan than elsewhere [9]. Physicians should know that α-GI-associated PI spontaneously resolves within 28 days of the discontinuation of the drug [9].

Many aspects of the pathogenic mechanism of PI remain unclear, and two major pathophysiological theories, the mechanical theory and the bacterial theory, have been proposed [1]. The mechanical theory suggests that PI is caused by increased luminal pressure, which forces air into the gastrointestinal tract wall through mucosal defects, whereas the infectious theory postulates that PI results from the accumulation of gas produced by aerogenic bacteria. The markedly elevated hydrogen concentration in the intramural gas is one line of evidence supporting the bacterial theory because hydrogen gas is a bacterial byproduct [1]. Moreover, the injection of *Clostridium perfringens*, *Escherichia coli*, or *Aerobacter aerogenes* into the bowel wall can induce PI in guinea pigs, whereas the injection of air or saline alone does not [10]. An experimental guinea-pig model of PI induced with *C. welchii* showed the effectiveness of a combination therapy of antibiotics and hyperbaric oxygen (HBO) therapy [8]. HBO therapy is also effective in human patients with PI, suggesting that HBO therapy may directly eliminate anaerobic gas-producing microorganisms [1]. Bacteria are rarely cultured from PI lesions, and only *Clostridium* spp., including *C. butyricum* and *C. parputrificum*, were detected in the PI lesions of neonates presenting with necrotizing enterocolitis [1, 11]. Although *C. difficile* can cause enteritis in the small intestine [12], *Clostridium* spp. inhabit the areas between the mucosal folds of the ascending colon, whereas regions of the central lumen are enriched with *Bacteroidaceae*, *Enterococcaceae*, and *Lactobacillaceae* [13]. Most *Clostridium* spp. usually maintain a commensal relationship with the host, except *C. perfringens*, *C. difficile*, and *C. tetani*, which can produce pathogenic toxins [13]. Recently, a pathohistological study showed a strong positive immunohistochemical signal for podoplanin, a transmembrane mucoprotein and specific marker of lymphatic endothelial cells, in the lining cells surrounding the inside of the intramural cystic spaces formed during PI, suggesting that PI involves gas-distended lymphatic vessels [14]. These findings suggest that the invasion of *Clostridium* spp. into the intestinal lymphatic vessels is associated with the onset of PI. However, this possibility prompts the question: why did our two patients show no signs of inflammation at or after admission? case 2 suffered α-GI-associated PI, and in this context, gas production may be increased by the fermentation of unabsorbed carbohydrates, and the hypoperistaltic status of the bowel that is associated with diabetes mellitus may contribute to the onset of PI [15]. Urita et al. [16] reported that small-intestinal bacterial overgrowth (SIBO) is potentially present in three-quarters of Japanese diabetic patients, suggesting that SIBO, exacerbated by the administration of α-GI, may have also been associated with the onset of PI in case 2, in whom PI mainly involved the small intestine. Most patients presenting with α-GI-associated PI can be managed with conservative treatment and follow a benign clinical course [15]. Therefore, the lack of any inflammatory response in patients with benign PI may indicate that an unknown mechanism inhibits the progression of bacterial infection and the consequent inflammatory response in the host.

The gastrointestinal tract is an organ that is always in contact with a variety of microbes and diverse pathogens. Various immune cells maintain a balance between the immunogenic and tolerogenic immune responses, and dendritic cells (DCs) and macrophages play particularly key roles in the host defenses against pathogens [17]. Interestingly, intestinal DCs have regulatory properties that mediate immunotolerance, and colonic DCs are better adapted to the greater bacterial load in the colon than are ileal DCs [18]. *Clostridium* spp. can also affect the development and function of regulatory T cells in the intestine, leading to tolerance in the gut microbiota through the induction of transforming growth factor-β in lamina propria DCs [13, 19]. A recent study demonstrated that *C. butyricum*, a Gram-positive anaerobe found in the intestines of healthy humans, regulates an anti-inflammatory response by activating interleukin-10-producing macrophages [20]. This suggests that benign PI is caused by commensal *Clostridium* spp., which play a key role in gut immunotolerance. The high incidence of benign PI in the colon, rather than the small intestine [7], may also reflect the immunosuppressive properties of colonic DCs, which are partly induced by *Clostridium* spp., leading to the absence of an inflammatory response. However, this is conjectural and the limitations of our study include our inability to endoscopically or histopathologically examine the PI lesions of our patients or to demonstrate the presence of *Clostridium* spp. in their PI lesions. Moreover, we could not analyze the intestinal flora in these patients in detail because the inspection capacity of the microbiology laboratory at our hospital is limited. Therefore, it is unclear whether certain bacteria were elevated or reduced in the intestinal flora of these patients.

## Conclusions

We treated two patients with benign PI, both of whom showed no marked inflammatory signs and were only treated with fasting and intravenous fluids. The mechanism of the pathogenesis of PI remains unclear. However, the features of our two patients with benign PI, especially the absence of an inflammatory response and the spontaneous resolution of their disease without specific treatment, suggest the participation of nonpathogenic indigenous bacteria of the gastrointestinal tract.

## Abbreviations

α-GI: α-glucosidase inhibitor
CRP: C-reactive protein
CT: computed tomography
DC: dendritic cell
HBO: hyperbaric oxygen
PI: pneumatosis intestinalis
SIBO: small-intestinal bacterial overgrowth
WBC: white blood cell
